# Enhancing surgical outcomes in dengue patients: strategic approaches to anesthetic management and timing of elective surgery

**DOI:** 10.1016/j.bjane.2024.844514

**Published:** 2024-05-13

**Authors:** Marina Ayres Delgado, Andre Luis Vieira Drumond, André dos Santos Mendonça, Camila Gomes Dall'Aqua, Márcio Erlei Vieira de Sá Filho, Bruno Pessoa Chacon, Lais Mendes Viana

**Affiliations:** a Universidade Federal de Minas Gerais, Hospital das Clínicas de Belo Horizonte, Divisão de Anestesiologia, Departamento de Cirurgia, Belo Horizonte, MG, Brazil; b Hospital da Polícia Militar, Belo Horizonte, MG, Brazil

Dear Editor,

Dengue fever remains a significant global health concern, particularly in tropical and subtropical regions where it manifests as a cyclical outbreak, affecting millions annually. The recent revisions to the WHO case classification in 2009 aimed to streamline diagnosis and management, yet challenges persist, especially concerning surgical interventions in dengue patients.^1^

Dengue can be detected in its early stages (less than five days) by virus isolation, RNA detection, or by looking for antigens like NS1. After this time (more than five days after infection), the viremia has decreased and antibody responses have developed, making dengue virus RNA and antigens potentially undetectable. At this point, serological techniques for specific antibody identification (IgM or IgG detection) are appropriate.^1^

Despite advancements in diagnostic techniques enabling early identification, the optimal timing for elective surgery post-dengue infection remains ambiguous. Furthermore, there is a notable absence of consensus on anesthetic management strategies tailored to dengue patients undergoing surgery. This critical gap underscores the urgent need for standardized protocols and guidelines in this domain to ensure optimal patient outcomes.^2^^,^^3^

Dengue's pathophysiology presents complexities for surgical interventions, particularly concerning coagulopathy and microvascular alterations observed in severe cases. Thromboelastographic (TEG) analyses of dengue patients have shown coagulation factor deficiency as the major abnormality followed by platelet dysfunction, primary fibrinolysis but sustained fibrinogen levels.^4^ Despite extensive search, it is important to note that the existing literature lacks definitive guidelines or recommendations regarding the optimal timing for elective surgery following dengue virus infection. Additionally, there is a paucity of guidance on the most effective anesthetic management during surgery in patients with dengue fever. This gap underscores the necessity for further research and the development of standardized protocols in these critical areas of patient care.^2^

Microvascular alterations and coagulopathy brought on by severe dengue may make surgical interventions dangerous and surgical management difficult. The gastrointestinal, hepatic, renal, hematological, neurological, respiratory, and cardiac systems are becoming more frequently involved in disease patterns. One recognizable symptom of dengue hemorrhagic fever during its crucial period is abdominal pain. While some dengue patients may simulate an acute abdomen without any real issues, others may present with surgical complications such as gastrointestinal hemorrhage, acute pancreatitis, and splenic rupture.^2^

Diagnosis of an acute abdomen can be difficult in a critically ill dengue patient and should be considered. Dengue infections may be associated with true or apparent surgical acute abdomen because of several reasons. A true acute abdomen may occur as a complication of dengue fever (ruptured splenic hematomas, upper and lower gastrointestinal bleeding, and abdominal wall hematomas). Dual pathologies may occur and can be related to dengue, for example, acute acalculous cholecystitis and appendicitis. Dengue infections may occur with other simultaneous surgical conditions unrelated to dengue (e.g., bowel obstruction). The pathophysiological mechanisms for acute abdomen in dengue are poorly understood. It may be because of direct viral invasion of the abdominal organs such as the appendix, gallbladder wall, pancreas, or spleen leading to inflammation and edema.

A systemic inflammatory response may be another reason. As most patients who presented acute abdomen had features of dengue hemorrhagic fever or dengue shock syndrome, the systemic inflammatory response and plasma leakage may have led to the edematous and inflammatory changes within organs. An edematous appendix with luminal obstruction may precipitate a secondary bacterial infection and cause appendicitis. The pathogenesis of acute acalculous cholecystitis may be multifactorial. The systemic inflammatory response, endotoxemia, cholestasis, secondary bacterial translocation, microangiopathic changes, and ischemia reperfusion injury may contribute.^2^ Edema of splenic parenchyma due to fluid leakage or bleeding with expansion of a hematoma within a non-yielding splenic capsule may cause spontaneous splenic rupture. The systemic inflammatory response with coagulopathy can lead to spontaneous bleeding or increased bleeding during minor trauma, especially when there is an underlying pathology such as mucosal ulceration.^2^

Anesthetic management in dengue patients poses unique challenges, primarily due to severe thrombocytopenia, coagulopathy, and vasculopathy, which heighten the risk of major bleeding and mortality during surgery. Noninvasive and invasive monitoring techniques are essential for maintaining hemodynamic stability, while special precautions are warranted during induction and intubation to minimize complications.^3^

Prophylactic platelet transfusions are discouraged due to associated risks, while alternative interventions such as cryoprecipitate and recombinant activated Factor VII may be considered in cases of life-threatening bleeding. Fluid resuscitation protocols must be tailored to address plasma leakage and shock, with emphasis placed on goal-directed therapy to optimize cardiac preload and organ perfusion.^4^

The current literature lacks definitive guidelines on the ideal interval for elective surgery post-dengue infection, with few centers adopting protocols based on disease severity and surgical complexity. A prior study has identified the optimal timeframe for renal transplant surgery in both donors and recipients who test positive for dengue. Patients with positive dengue screening should undergo a waiting period averaging 6 weeks and must test negative for NS1.^5^

Complete clinical and laboratory recovery, including normalization of blood counts, liver function, renal parameters, and coagulation profiles, should precede elective procedures.

Prospective, randomized-controlled trials exploring anesthetic management during surgery in dengue patients and preoperative protocols for those with prior dengue virus infection are urgently needed. Although multicentric studies with robust follow-up mechanisms hold promise for elucidating optimal practices in this complex clinical scenario and represent the gold standard of evidence, it appears that they may not be currently feasible, particularly given the small number of dengue fever patients who will require surgical procedures.

The literature on anesthesia management in patients with dengue fever is mainly in the form of case reports or case series. Such studies could help establish evidence-based guidelines, improve surgical outcomes in this complex clinical scenario, and investigate the impact of different anesthetic approaches or tailored preoperative protocols. That could offer valuable insights into enhancing outcomes for dengue patients undergoing surgery.

For instance, Joshi et al highlight the challenges of managing anesthesia in pregnant dengue patients for emergency cesarean section and underscore the need for tailored strategies.^6^ Additionally, Hariyanto et al emphasize the importance of TEG as a point-of-care test, which may clearly identify the coagulation abnormalities in dengue fever patients with hemorrhagic complications. The authors suggest a TEG-based therapeutic strategy, in order to avoid potential complications related to unnecessary transfusions in these patients.^7^

Delving deeper into these interventions and rationales is crucial. Future research, such as multicentric studies with robust follow-ups mechanisms to track the long-term outcomes of dengue patients undergoing surgery, could focus on elucidating the optimal timing and dosage of anesthesia in these patients, as well as developing standardized preoperative protocols tailored to their unique needs. The efforts would not only improve patient outcomes, but also contribute significantly to the existing body of knowledge on surgical management in dengue patients.

In conclusion, addressing the challenges associated with surgical interventions in dengue patients requires interdisciplinary collaboration and evidence-based protocols. By prioritizing research efforts in this domain, we can enhance patient care and minimize perioperative complications in this vulnerable population.

## Conflicts of interest

The authors declare no conflicts of interest.

## References

[1] Harapan H, Michie A, Sasmono RT, Imrie A. Dengue: A Minireview. 2020;1:1-35. 10.3390/v12080829 PMC7472303 32751561

[2] Jayarajah U, Lahiru M, de Zoysa I, Seneviratne SL. (2021). Review article dengue infections and the surgical patient. Am J Trop Med Hyg.

[3] Tovar JH, Pinzón MA, Rincón DF, Jiménez-Canizalez CE, Mondragón-Cardona Á, Arrieta-Mendoza ME (2018). Anesthetic considerations in the patient with dengue virus disease. Rev Chil Anest.

[4] Chhabra A, Malhotra N. (2006). Anesthetic management of a pregnant patient with dengue hemorrhagic fever for emergency cesarean section. Int J Obstet Anesth.

[5] Etta PK. (2020). Screening for dengue virus in endemic areas prior to transplantation: Indian perspective. Indian J Transplant.

[6] Joshi N, Gupta L, Chatterjee R. (2023). Anaesthetic management of pregnant patients with severe dengue fever for emergency caesarean section ‒ A case series and review. Indian J Clin Anaesth.

[7] Hariyanto H, Yahya CQ, Wibowo P, Tampubolon OE. (2016). Management of severe dengue hemorrhagic fever and bleeding complications in a primigravida patient: A case report. J Med Case Rep.

